# Supplementary material for: Antibodies utilizing VL6-57 light chains target a convergent cryptic epitope on SARS-CoV-2 spike protein and potentially drive the genesis of Omicron variants
Source: Nat Commun. 2024 Aug 31;15:7585. doi: 10.1038/s41467-024-51770-3 (PMC11366018; doi:10.1038/s41467-024-51770-3)
Supplement: Supplementary file 1 — Supplementary Information [file 41467_2024_51770_MOESM1_ESM.pdf]

Supplementary Materials

Antibodies utilizing VL6-57 light chains target a convergent cryptic epitope on SARS-CoV-2 spike protein and potentially drive the genesis of Omicron variants

Supplementary Information includes:

Supplementary Figures 1-11
Supplementary Tables 1-4
Supplementary References

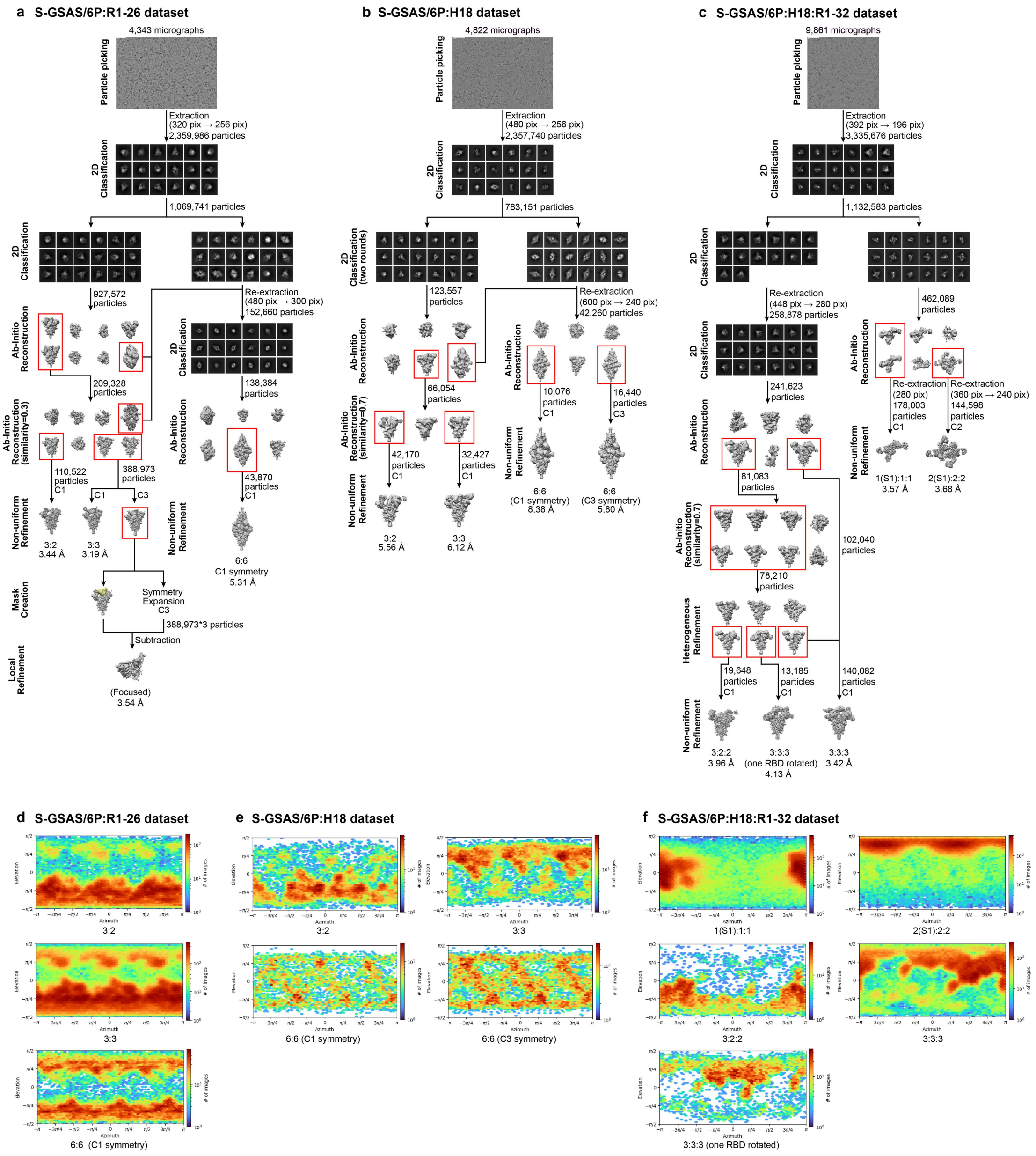

Fig. S1 | Cryo-EM data processing pipelines. a, Data processing pipeline for the S-GSAS/6P:R1-26 complex dataset, the mask for subtraction is colored in yellow. **b**, Data processing pipeline for the S-GSAS/6P:H18 complex dataset. **c**, Data processing pipeline for the S-GSAS/6P:H18:R1-32 complex dataset. Red boxes indicate selected classes. **d**,**e**,**f**, Angular distribution of the particles used for the reconstructions.

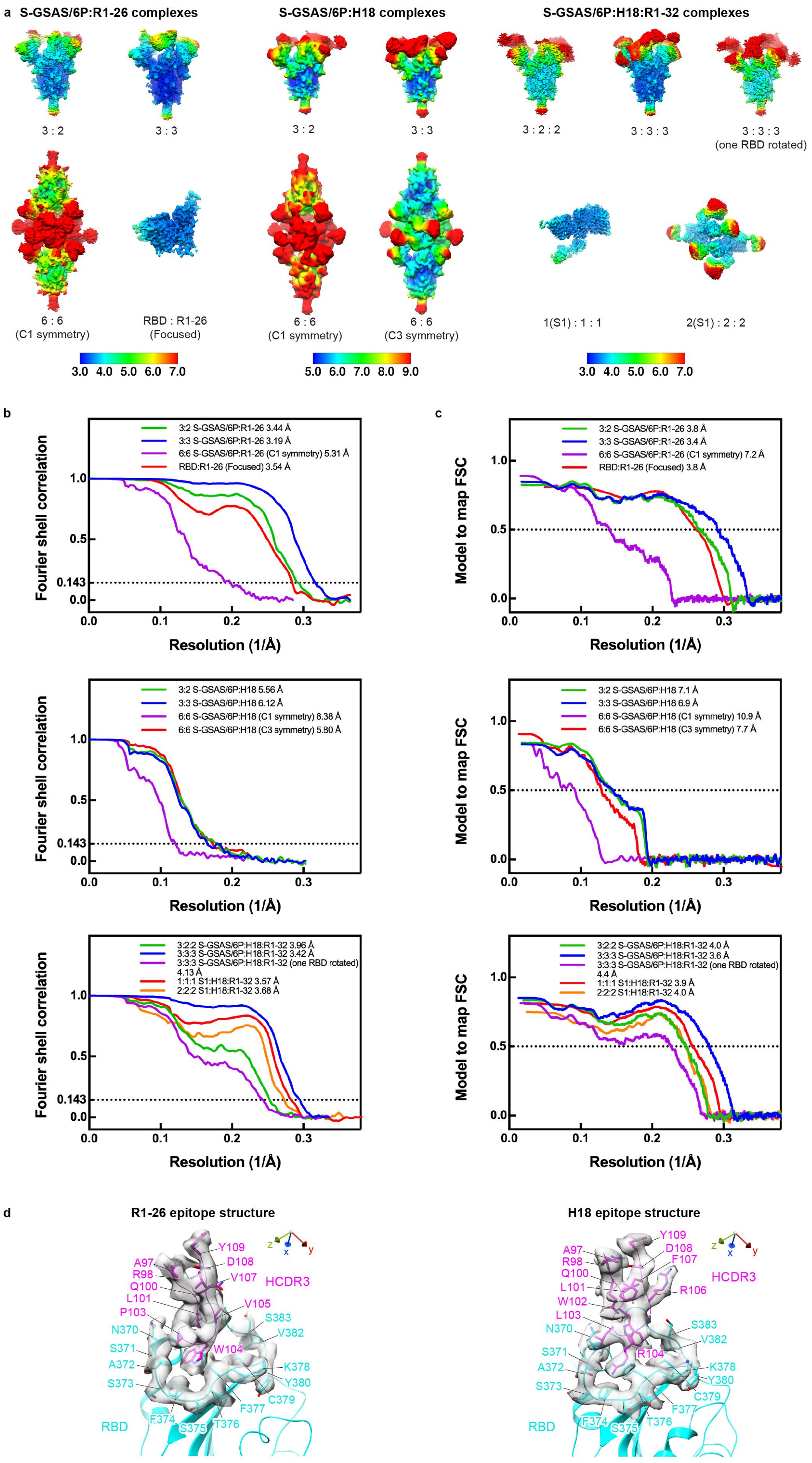

a
S-GSAS/6P:R1-26 complexes
S-GSAS/6P:H18 complexes
S-GSAS/6P:H18:R1-32 complexes
3 : 2
3 : 3
3 : 2
3 : 3
3 : 2 : 2
3 : 3 : 3
3 : 3 : 3
(one RBD rotated)
6 : 6
(C1 symmetry)
RBD : R1-26
(Focused)
6 : 6
(C1 symmetry)
6 : 6
(C3 symmetry)
1(S1) : 1 : 1
2(S1) : 2 : 2
3.0 4.0 5.0 6.0 7.0
5.0 6.0 7.0 8.0 9.0
3.0 4.0 5.0 6.0 7.0
b
c
Fourier shell correlation
Model to map FSC
Resolution (1/Å)
3:2 S-GSAS/6P:R1-26 3.44 Å
3:3 S-GSAS/6P:R1-26 3.19 Å
6:6 S-GSAS/6P:R1-26 (C1 symmetry) 5.31 Å
RBD:R1-26 (Focused) 3.54 Å
3:2 S-GSAS/6P:R1-26 3.8 Å
3:3 S-GSAS/6P:R1-26 3.4 Å
6:6 S-GSAS/6P:R1-26 (C1 symmetry) 7.2 Å
RBD:R1-26 (Focused) 3.8 Å
3:2 S-GSAS/6P:H18 5.56 Å
3:3 S-GSAS/6P:H18 6.12 Å
6:6 S-GSAS/6P:H18 (C1 symmetry) 8.38 Å
6:6 S-GSAS/6P:H18 (C3 symmetry) 5.80 Å
3:2 S-GSAS/6P:H18 7.1 Å
3:3 S-GSAS/6P:H18 6.9 Å
6:6 S-GSAS/6P:H18 (C1 symmetry) 10.9 Å
6:6 S-GSAS/6P:H18 (C3 symmetry) 7.7 Å
3:2:2 S-GSAS/6P:H18:R1-32 3.96 Å
3:3:3 S-GSAS/6P:H18:R1-32 3.42 Å
3:3:3 S-GSAS/6P:H18:R1-32 (one RBD rotated) 4.13 Å
1:1:1 S1:H18:R1-32 3.57 Å
2:2:2 S1:H18:R1-32 3.68 Å
3:2:2 S-GSAS/6P:H18:R1-32 4.0 Å
3:3:3 S-GSAS/6P:H18:R1-32 3.6 Å
3:3:3 S-GSAS/6P:H18:R1-32 (one RBD rotated) 4.4 Å
1:1:1 S1:H18:R1-32 3.9 Å
2:2:2 S1:H18:R1-32 4.0 Å
d
R1-26 epitope structure
H18 epitope structure
HCDR3
RBD
A97
R98
Q100
L101
P103
W104
V105
V107
D108
Y109
N370
S371
A372
S373
F374
S375
T376
F377
K378
C379
Y380
V382
S383
W102
L103
R104
R106
F107

Fig. S2 | Resolution assessment of cryo-EM structures. a, Local resolution assessment for the complex structures determined from S-GSAS/6P:R1-26, S-GSAS/6P:H18 and S-GSAS/6P:H18:R1-32 datasets. **b**, Global resolution assessment by Fourier shell correlation (FSC) at the 0.143 criterion. **c**, Correlations of model vs map by FSC at the 0.5 criterion. **d**, Representative cryo-EM densities around antibody-RBD binding interfaces. HCDR3 loops of R1-26 or H18 are colored magenta and RBD is colored cyan.

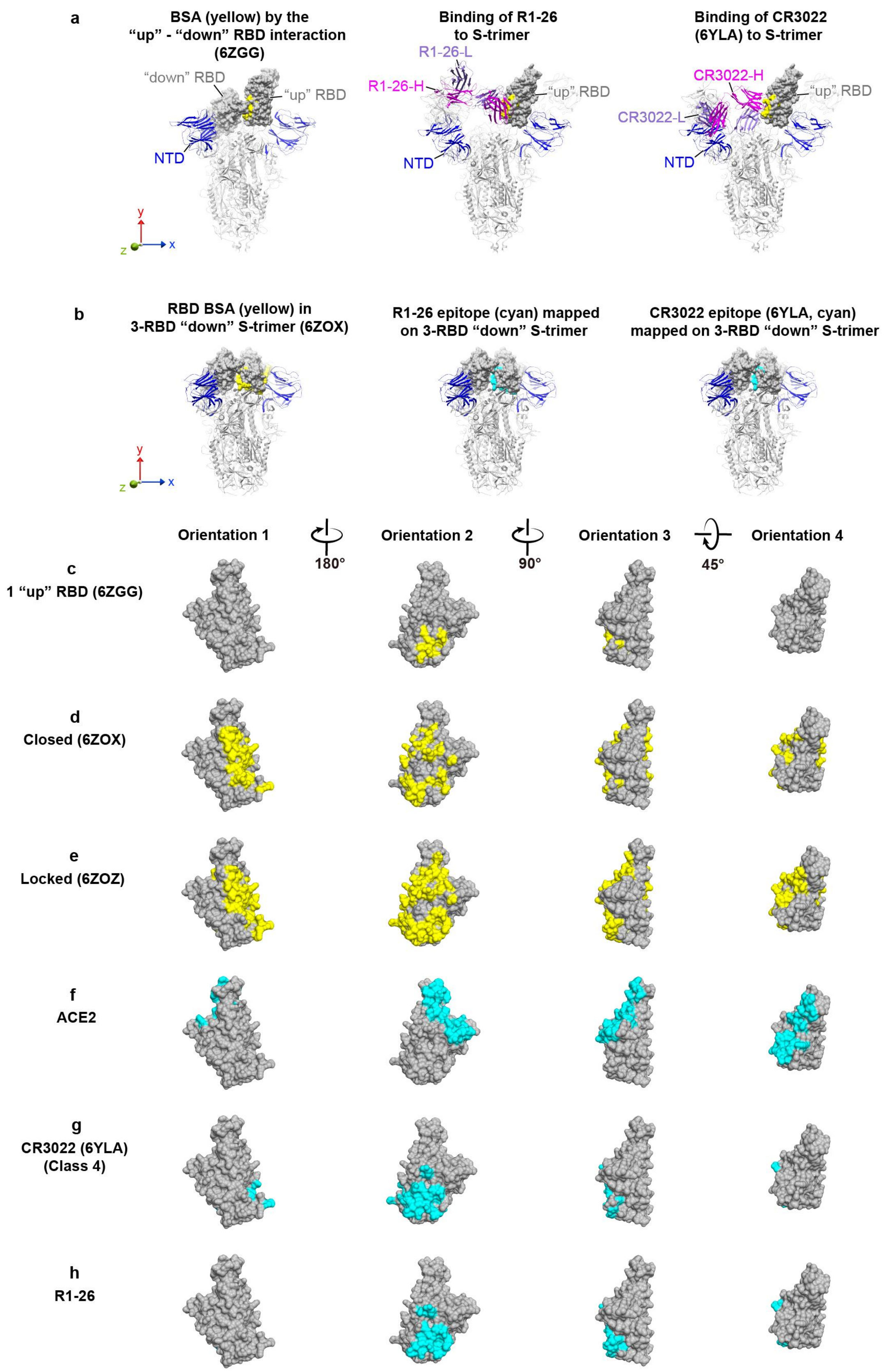

Fig. S3 | Epitope of R1-26 in the context of different S-trimer conformations and compared to other buried surface areas (BSAs)

on RBD. RBD molecular surfaces are colored in gray, BSAs in different S-trimer conformations are highlighted in yellow, BSAs by ACE2 or antibodies are highlighted in cyan. **a**, In each of the shown 1 RBD “up” S-trimers[1], buried surface area on the “up” RBD buried by the adjacent “down” RBD is colored yellow (also shown in **c**). Binding of R1-26 (middle) or CR3022 (right)[2] is shown and compared with the buried surface area by the “down” RBD. **b**, In each of the shown 3 RBD “down” closed S-trimer structures[3], buried RBD surface area (left, colored yellow, also shown in **d**) or epitope of R1-26 (middle, cyan, also shown in **h**) or CR3022 (right, cyan, also shown in **g**) is shown to illustrate R1-26 and CR3022 epitopes being cryptic. **e**, RBD buried surface area in the 3 RBD “down” locked S-trimer structure[3]. **f-h**, Binding surface of ACE2 (**f**)[4] and epitope of CR3022 (**g**) or R1-26 (**h**).

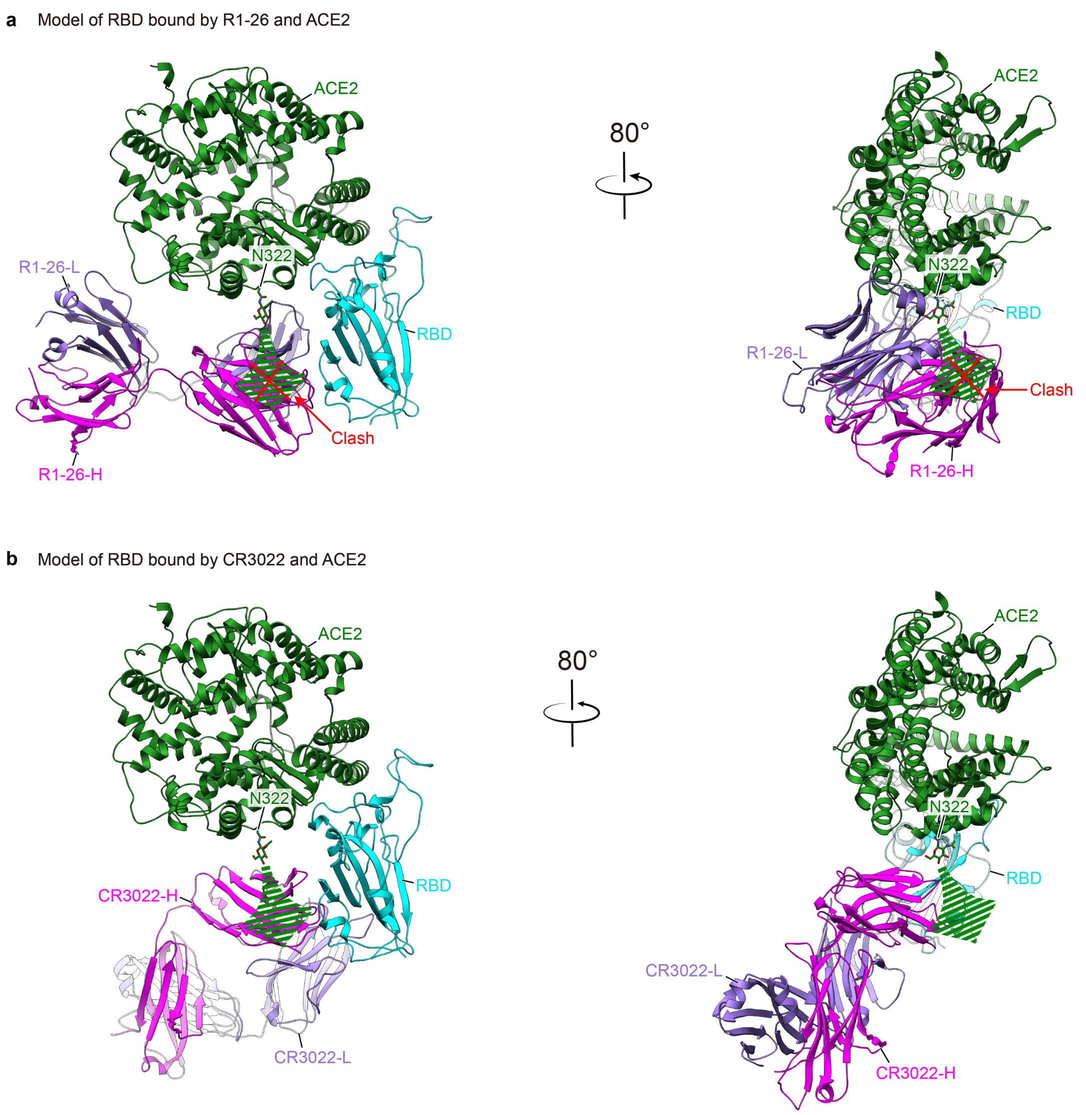

Fig. S4 | A hypothetical model showing simultaneous RBD binding by ACE2 and mAb R1-26 or CR3022. a, A hypothetical model showing simultaneous RBD binding by R1-26 and ACE2. The model is based on RBD complex structures of R1-26 and ACE2 (PDB: 7YDI). The glycan attached to ACE2 residue N322 is labelled and the green dashed arrow indicates a possible direction of glycan chain extension. **b**, A hypothetical model showing simultaneous RBD binding by CR3022 and ACE2. The model is based on RBD complex structures of CR3022 (PDB: 6W41) and ACE2 (PDB: 7YDI). The glycan attached to ACE2 residue N322 is labelled and the green dashed arrow indicates a possible direction of glycan chain extension.

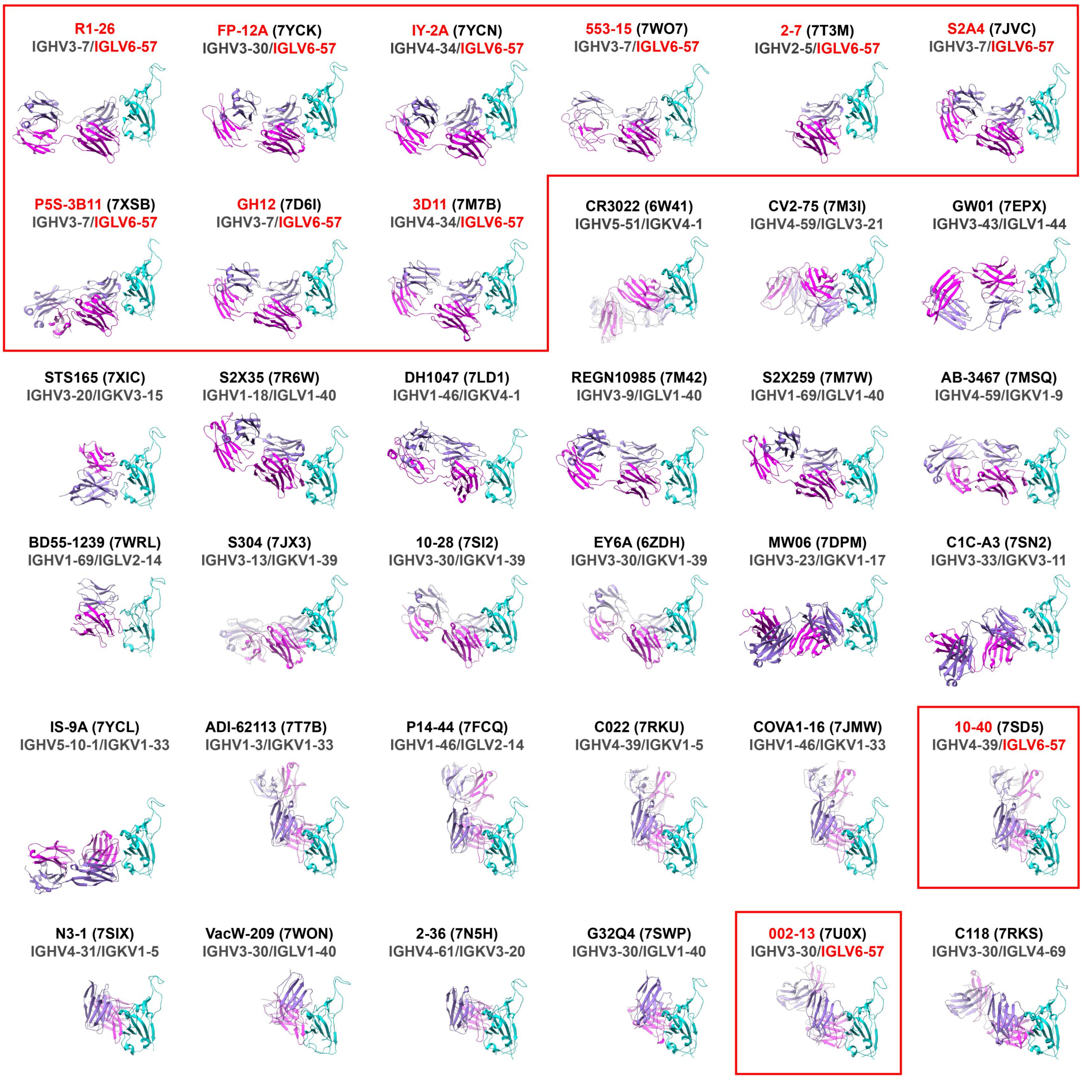

Fig. S5 | Structure gallery of class 4 antibodies in complex with RBD. Antibody H chains and L chains are colored in magenta and purple. RBDs are colored in cyan. Antibody heavy and light chain V genes encoding each antibody are shown under each antibody name with corresponding PDB code in backet. IGLV6-57 encoded antibody structures are highlighted by their names and light chain V genes shown in red. Structures are arranged by similarity of their binding modes.

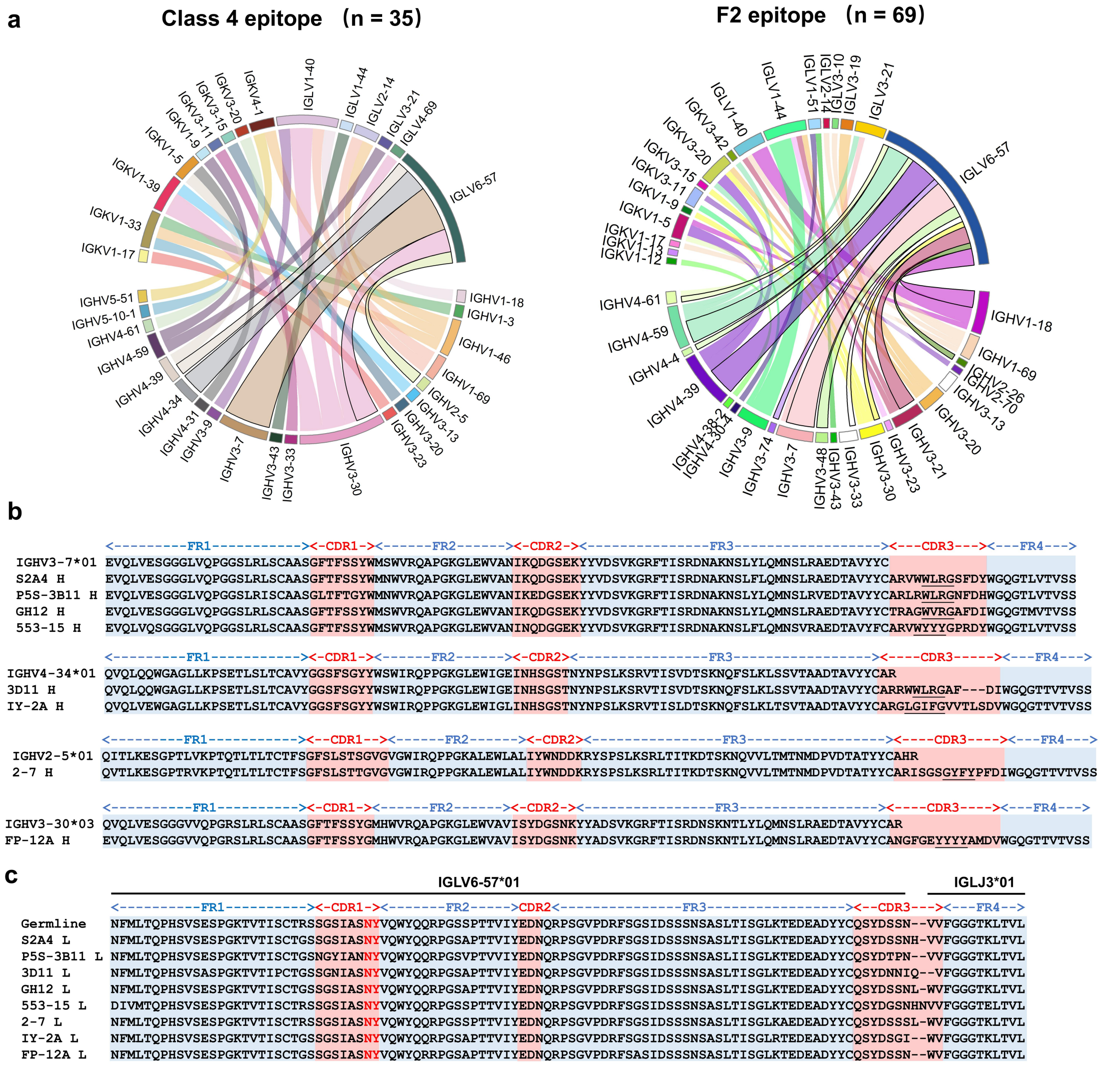

Fig. S6 | Sequence analysis of class 4 mAbs and VL6-57 utilizing mAbs. a, Germline heavy and light chain gene usage and pairing among the 35 class 4 (left) and the 69 class F2 (right) antibodies. In the chord diagrams, each of the germline heavy and light chain pair is linked by an arc, the size of arc is proportional to the number of antibodies identified with such heavy-light chain pairing. **b**, Heavy chain sequence alignment of the 8 structurally characterized VL6-57 utilizing mAbs with their germline gene sequences. Hydrophobic, particularly aromatic amino acid residues within HCDR3 regions are underlined. **c**, Light chain sequence alignment of the 8 structurally characterized VL6-57 utilizing mAbs with their germline gene sequences. The RBD-contact motif “NY” encoded by VL6-57 light chain LCDR1 germline sequence is shown in red letters.

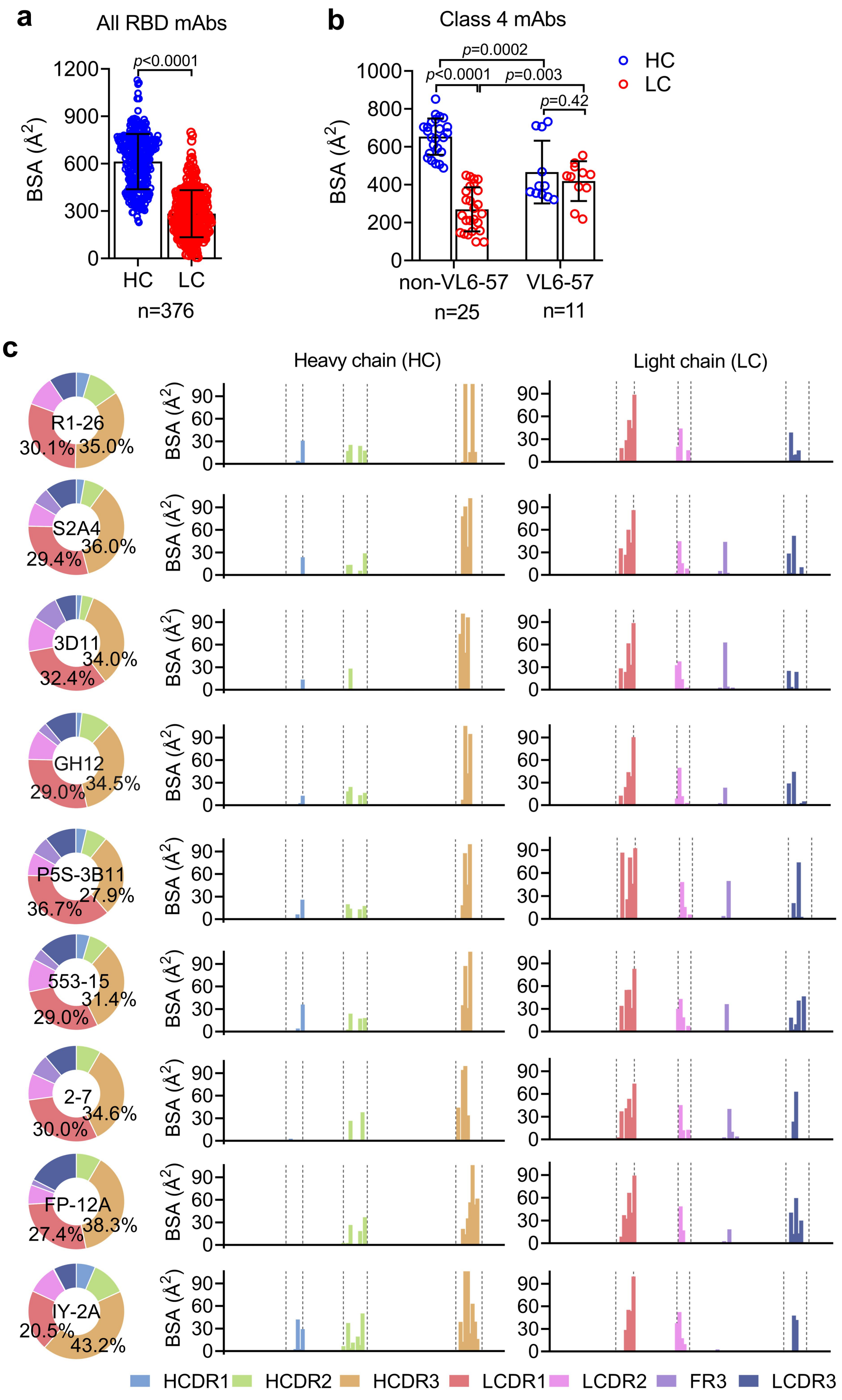
a
All RBD mAbs
p<0.0001
BSA (Å2)
1200
900
600
300
0
HC
LC
n=376
b
Class 4 mAbs
p=0.0002
p<0.0001
p=0.003
p=0.42
1000
800
600
400
200
0
non-VL6-57
VL6-57
n=25
n=11
HC
LC
c
Heavy chain (HC)
Light chain (LC)
R1-26
30.1%
35.0%
S2A4
29.4%
36.0%
3D11
32.4%
34.0%
GH12
29.0%
34.5%
P5S-3B11
36.7%
27.9%
553-15
29.0%
31.4%
2-7
30.0%
34.6%
FP-12A
27.4%
38.3%
IY-2A
20.5%
43.2%
90
60
30
0
HCDR1
HCDR2
HCDR3
LCDR1
LCDR2
FR3
LCDR3

Fig. S7 | Buried surface area (BSA) analysis of the structurally characterized spike-specific mAbs. a, Comparison of BSAs by heavy (HC) and light chains (LC) of the 376 structurally characterized spike-specific mAbs available in the PDB. **b**, Differences in BSAs by heavy (HC) and light chain (LC) are compared between VL6-57 (R1-26 is included in this analysis) and non-VL6-57 class 4 mAbs. **c**, Per-site BSA analysis on heavy chains (HC) and light chains (LC) of R1-26 and the other 8 VL6-57 mAbs. Data are presented as mean values ± SD, and two-tailed unpaired student's t test was performed in panels (**a**) and (**b**). Source data for (**a**) and (**b**) are provided as a Source Data file.

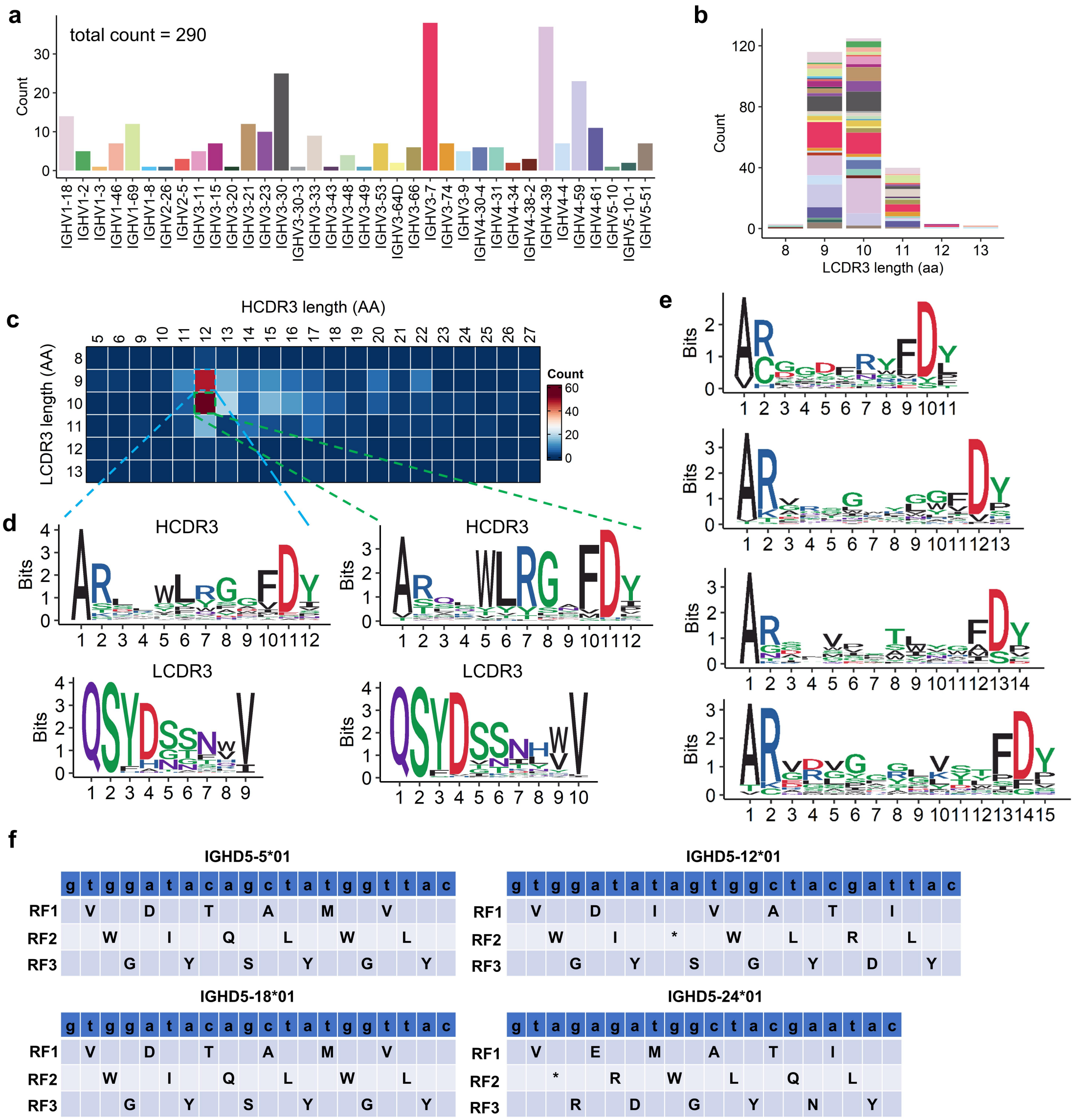

Fig. S8 | Sequence analysis of VL6-57 utilizing mAbs from the CoV-AbDab. a, A histogram showing the heavy chain germline gene usage of the 290 VL6-57 utilizing mAbs curated from the CoV-AbDab. **b**, A histogram showing the LCDR3 length distribution of the 290 VL6-57 utilizing mAbs. **c**, A heatmap showing the HCDR3 and LCDR3 length pairing and distribution among the 290 VL6-57 utilizing mAbs. **d**, Sequence logo plots showing consensus amino acids within 12-aa length HCDR3 loops and their paired 9-aa (left) or 10-aa (right) length LCDR3 loops of VL6-57 mAbs. **e**, Sequence logo plots showing consensus amino acids of HCDR3 loops of 11-, 13-, 14-, or 15-aa length among the 290 VL6-57 utilizing mAbs. **f**, Three reading frames (RF1-3) of IGHD5

germline genes are shown. The “WLR” sequence is encoded by RF2 of IGHD5-12 (top right).

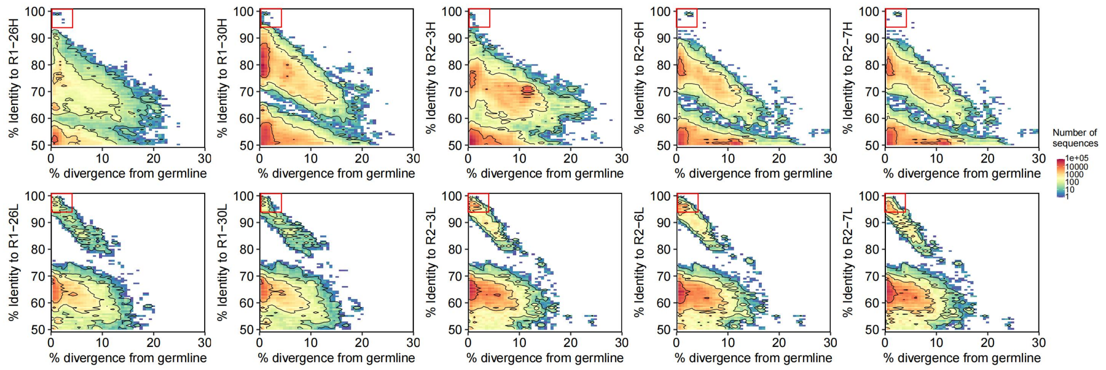

Fig. S9 | Identity-divergence plots showing the presence of R1-26, R1-30, R2-3, R2-6, or R2-7-like IgH or IgL lineages in the repertoires of their donors. All sequences in the repertoires are plotted as a function of sequence divergence from their germline genes (x axis) and sequence identity to heavy chain or light chain of R1-26, R1-30, R2-3, R2-6, or R2-7 (y axis). Color gradient indicates sequence density. Red boxes indicate peaks containing IgH or IgL sequences highly similar to those of R1-26, R1-30, R2-3, R2-6, or R2-7.

a

mAb Id	Heavy chain: IGHV	IGHD	IGHJ	HCDR3	HCDR3 length	SHM%	Light chain: IGLV	IGLJ	LCDR3	LCDR3 length	SHM%	Origin of donor
H4	IGHV3-7	IGHD5-18	IGHJ3	ASQLWLRGAFDI	12	0						Healthy donor H4
H5	IGHV3-11	IGHD5-18	IGHJ4	ARAKWLRGHFDY	12	0						Healthy donor H5
H14	IGHV4-31	IGHD5-18	IGHJ3	ARKGWLRGAFDI	12	0	IGLV6-57	IGLJ3	QSYDSSNVV	9	0	Healthy donor H14
H16	IGHV4-39	IGHD5-12	IGHJ4	ARREWLRGPFDY	12	0						Healthy donor H16
H18	IGHV4-61	IGHD5-18	IGHJ4	ARQLWLRGRFDY	12	1.3						Healthy donor H18

b

```
                                IGHV3-7*01                                                          IGHD5-18*01  IGHJ3*02
Germline EVQLVESGGGLVQPGGSLRLSCAASGFTFSSYWMSWVRQAPGKGLEWVANIKQDGSEKYYVDSVKGRFTISRDNAKNSLYLQMNSLRAEDTAVYYCARQLWL--AFDIWGQGTMVTVSS
H4 H     ................................................................................................ .S....RG.....................

                                IGHV3-11*01                                                         IGHD5-18*01  IGHJ4*01
Germline QVQLVESGGGLVKPGGSLRLSCAASGFTFSDYYMSWIRQAPGKGLEWVSYISSSGSTIYYADSVKGRFTISRDNAKNSLYLQMNSLRAEDTAVYYCAR--WL--HFDYWGQGTLVTVSS
H5 H     ..................................................................................................AK..RG...............

                                IGHV4-31*01                                                         IGHD5-18*01  IGHJ3*02
Germline QVQLQESGPGLVKPSQTLSLTCTVSGGSISSGGYYWSWIRQHPGKGLEWIGYIYYSGSTYYNPSLKSLVTISVDTSKNQFSLKLSSVTAADTAVYYCAR--WL--AFDIWGQGTMVTVSS
H14 H    ...................................................................................................KG..RG...............

                                IGHV4-39*01                                                         IGHD5-12*01  IGHJ4*02
Germline QLQLQESGPGLVKPSETLSLTCTVSGGSISSSSYYWGWIRQPPGKGLEWIGSIYYSGSTYYNPSLKSRVTISVDTSKNQFSLKLSSVTAADTAVYYCAR--WLR--FDYWGQGTLVTVSS
H16 H    ...................................................................................................RE...GP..............

                                IGHV4-61*08                                                         IGHD5-18*01  IGHJ4*02
Germline QVQLQESGPGLVKPSETLSLTCTVSGGSVSSGGYYWSWIRQPPGKGLEWIGYIYYSGSTNYNPSLKSRVTISVDTSKNQFSLKLSSVTAADTAVYYCARQLWL---FDYWGQGTLVTVSS
H18 H    ..................................F................C.........................D...................RGR..............
```

c

```
                                IGLV6-57*02                                                         IGLJ3*01
         <----------FR1---------><-CDR1-><-------FR2-----><CDR2><-------------FR3----------------><--CDR3-><---FR4-->
Germline NFMLTQPHSVSESPGKTVTISCTRSSGSIASNYVQWYQQRPGSSPTTVIYEDNQRPSGVPDRFSGSIDSSSNSASLTISGLKTEDEADYYCQSYDSSNVVFGGGTKLTVL
Common L ..............................................................................................................
```

d

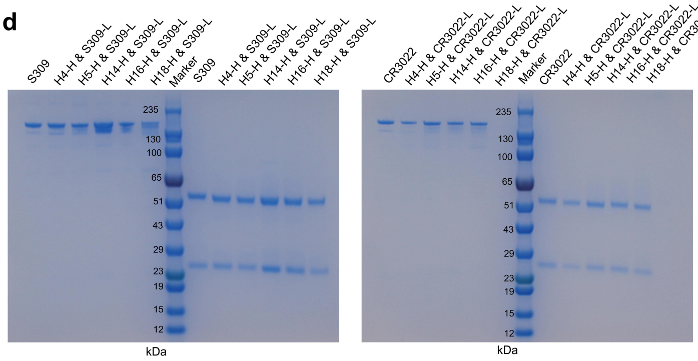

e

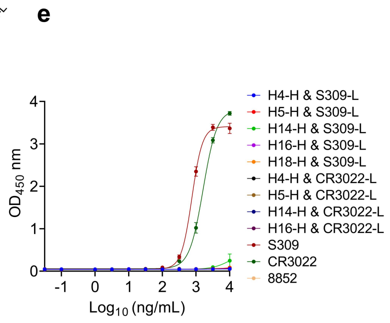

Fig. S10 | Characteristics of the recombinant **mAbs derived from SARS-CoV-2 naïve donors. a**, Summary of genetic features of the 5 recombinant mAbs derived from naïve donors. Sequence analysis of the mAbs was performed using IMGT/V-QUEST. **b**, Heavy chain sequence alignments of the 5 recombinant mAbs with their germline gene sequences. Amino acid residues identical to their germline sequence are dotted. **c**, Sequence alignment of the common light chain shared by the 5 recombinant mAbs with its germline gene sequence. **d**, SDS-PAGE showing the expression of the recombinant mAbs derived from the pairing of heavy chain of H4, H5, H14, H16 and H18 with the light chains of S309 (left) and CR3022 (right). **e**, ELISA binding assay for the recombinant mAbs derived from the pairing of heavy chain of H4, H5, H14, H16 and H18 with the light chains of S309 and CR3022 (data are presented as mean values ± SD, n=3). Source data for (**d**) and (**e**) are provided as a Source Data file.

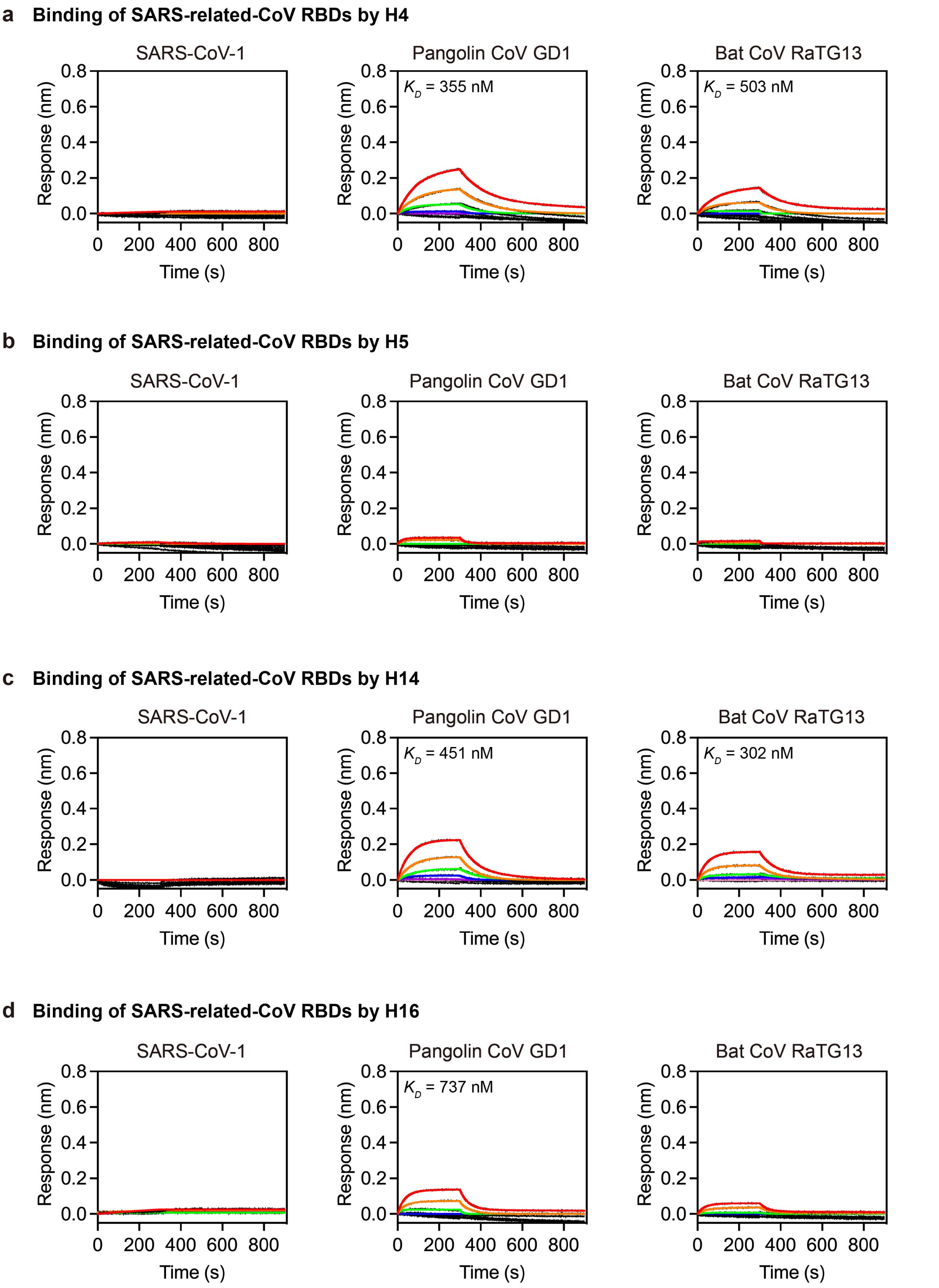

Fig. S11 | SARS-related-CoV RBD binding by the recombinant mAbs. **a-d**, Binding of H4 (**a**), H5 (**b**), H14 (**c**) and H16 (**d**) to SARS-CoV-1, Pangolin CoV GD1, and Bat CoV RaTG13 RBDs were measured by BLI. 2-fold serially diluted RBD solutions (200 nM to 3.125 nM) were used in

the BLI assays. Kinetic parameters are summarized in **Supplementary Table 3**.

Supplementary Table 1 | Summary of rate constants (*k*on, *k*off) and dissociation constants (K_D) for the R1-26 interactions in Fig. 1c, d

Ligand	Analyte	k_{on} (M^{-1} s^{-1})	k_{off} (s^{-1})	K_D (nM)
R1-26	RBD (WT)	4.60´ 10^{5}	1.77´ 10^{-3}	3.84
	RBD (Alpha)	6.25´ 10^{5}	6.90´ 10^{-3}	11.05
	RBD (Beta)	5.39´ 10^{5}	5.27´ 10^{-3}	9.78
	RBD (Delta)	3.41´ 10^{5}	3.51´ 10^{-3}	10.29
	RBD (Omicron BA.1)	-	-	no binding
	S-trimer (WT)	3.80´ 10^{3} (k_{on1})	1.37´ 10^{-6} (k_{off1})	0.36 (k_{off1}/k_{on1})
		2.38´ 10^{4} (k_{on2})	＜1.00´ 10^{-7} (k_{off2})	⩽0.058 (k_{off1}/k_{on2})
	S-trimer (Alpha)	1.08´ 10^{4} (k_{on1})	3.27´ 10^{-5} (k_{off1})	3.04 (k_{off1}/k_{on1})
		6.65´ 10^{4} (k_{on2})	2.29´ 10^{-7} (k_{off2})	0.49 (k_{off1}/k_{on2})
				⩽0.021 (k_{off2}/k_{on1})
	S-trimer (Beta)	1.21´ 10^{4} (k_{on1})	1.48´ 10^{-5} (k_{off1})	1.22 (k_{off1}/k_{on1})
		2.69´ 10^{4} (k_{on2})	＜1.00´ 10^{-7} (k_{off2})	0.55 (k_{off1}/k_{on2})
				＜0.0083 (k_{off2}/k_{on1})
	S-trimer (Delta)	1.71´ 10^{4} (k_{on1})	2.95´ 10^{-6} (k_{off1})	0.17 (k_{off1}/k_{on1})
		2.47´ 10^{4} (k_{on2})	＜1.00´ 10^{-7} (k_{off2})	0.12 (k_{off1}/k_{on2})
				＜0.0059 (k_{off2}/k_{on1})
	S-trimer (Omicron BA.1)	5.29´ 10^{3} (k_{on1})	2.54´ 10^{-4} (k_{off1})	47.99 (k_{off1}/k_{on1})
		9.09´ 10^{3} (k_{on2})	1.46´ 10^{-4} (k_{off2})	27.94 (k_{off1}/k_{on2})
				27.64 (k_{off2}/k_{on1})
				16.09 (k_{off2}/k_{on2})

Supplementary Table 2 | Cryo-EM data collection, refinement and validation statistics of S-GSAS/6P:R1-26, S-GSAS/6P:H18 and S-GSAS/6P:H18:R1-32

	S-GSAS/6P: R1-26 3:2 (EMD-60099, PDB 8ZHD)	S-GSAS/6P: R1-26 3:3 (EMD-60100, PDB 8ZHE)	S-GSAS/6P: R1-26 6:6 C1 symmetry (EMD-60101, PDB 8ZHF)	RBD:R1-26 /Focused (EMD-60102, PDB 8ZHG)	S-GSAS/6P: H18 3:2 (EMD-60103, PDB 8ZHH)
**Data collection and processing**					
Magnification	81,000	81,000	81,000	81,000	45,000
Voltage (kV)	300	300	300	300	200
Electron exposure ($e^{-}/Å^2$)	50	50	50	50	60
Defocus range (μm)	0.8-2.0	0.8-2.0	0.8-2.0	0.8-2.0	0.8-2.5
Pixel size (Å)	1.095	1.095	1.095	1.095	0.88
Movies (no.)	4,343	4,343	4,343	4,343	4,822
Initial particle images (no.)	2,359,986	2,359,986	2,359,986	2,359,986	2,357,740
Symmetry imposed	*C1*	*C1*	*C1*	*C1*	*C1*
Final particle images (no.)	110,522	388,973	43,870	1,166,919	42,170
Map resolution (Å)	3.44	3.19	5.31	3.54	5.56
FSC threshold	0.143	0.143	0.143	0.143	0.143
Map resolution range (Å)	3.16-11.51	2.99-10.63	4.09-19.19	3.38-5.72	4.40-19.60
**Refinement**					
Initial model used	PDB 7YEG	PDB 7YEG	PDB 7YEG	PDB 7YEG	PDB 7YEG
Model resolution (Å)	3.8	3.4	7.2	3.8	7.1
FSC threshold	0.5	0.5	0.5	0.5	0.5
Map sharpening *B* factor ($Å^2$)				-112	
Model composition					
Non-hydrogen atoms	32,311	35,558	70,965	3,310	32,374
Protein residues	3,656	4,503	8,985	425	4,072
Ligands	60	60	120	2	57
B factors ($Å^2$)					
Protein	302.63	301.53	367.59	143.06	526.83
Ligand	209.21	194.03	311.95	141.06	326.68
R.m.s. deviations					
Bond lengths (Å)	0.003	0.003	0.003	0.004	0.002
Bond angles (°)	0.633	0.611	0.609	0.758	0.548
**Validation**					
MolProbity score	1.60	1.61	1.62	1.60	1.53
Clashscore	5.76	5.98	5.79	5.13	5.85
Poor rotamers (%)	0.60	0.70	0.37	0.84	0.08
Ramachandran plot					
Favored (%)	95.82	95.91	95.59	95.23	96.67
Allowed (%)	4.18	4.09	4.41	4.77	3.33
Disallowed (%)	0.00	0.00	0.00	0.00	0.00

	S-GSAS/6P: H18 3:3 (EMD-60104, PDB 8ZHI)	S-GSAS/6P: H18 6:6 C1 symmetry (EMD-60105, PDB 8ZHJ)	S-GSAS/6P: H18 6:6 C3 symmetry (EMD-60106, PDB 8ZHK)	S-GSAS/6P: H18:R1-32 3:2:2 (EMD-60107, PDB 8ZHL)	S-GSAS/6P: H18:R1-32 3:3:3 (EMD-60108, PDB 8ZHM)
**Data collection and processing**					
Magnification	45,000	45,000	45,000	130,000	130,000
Voltage (kV)	200	200	200	300	300
Electron exposure ($e^{-}/Å^2$)	60	60	60	50	50
Defocus range (μm)	0.8-2.5	0.8-2.5	0.8-2.5	0.6-2.0	0.6-2.0
Pixel size (Å)	0.88	0.88	0.88	0.93	0.93
Movies (no.)	4,822	4,822	4,822	9,861	9,861
Initial particle images (no.)	2,357,740	2,357,740	2,357,740	3,335,676	3,335,676
Symmetry imposed	*C1*	*C1*	*C3*	*C1*	*C1*
Final particle images (no.)	32,427	10,076	16,440	19,648	140,082
Map resolution (Å)	6.12	8.38	5.80	3.96	3.42
FSC threshold	0.143	0.143	0.143	0.143	0.143
Map resolution range (Å)	4.62-19.17	6.69-24.88	4.49-19.01	3.58-19.47	3.18-12.85
**Refinement**					
Initial model used	PDB 7YEG	PDB 7YEG	PDB 7YEG	PDB 7YEG	PDB 7YEG
Model resolution (Å)	6.9	10.9	7.7	4.0	3.6
FSC threshold	0.5	0.5	0.5	0.5	0.5
Map sharpening *B* factor ($Å^2$)					
Model composition					
Non-hydrogen atoms	35,629	70,986	71,271	38,806	45,309
Protein residues	4,508	8,980	9,018	4,950	5,829
Ligands	57	114	114	57	57
B factors ($Å^2$)					
Protein	312.67	538.26	349.39	340.88	390.49
Ligand	190.03	503.00	335.34	190.03	190.03
R.m.s. deviations					
Bond lengths (Å)	0.003	0.002	0.003	0.004	0.002
Bond angles (°)	0.546	0.542	0.611	0.967	0.537
**Validation**					
MolProbity score	1.62	1.52	1.60	1.62	1.60
Clashscore	6.42	5.38	6.29	6.68	6.29
Poor rotamers (%)	0.71	0.09	0.20	0.59	0.76
Ramachandran plot					
Favored (%)	96.12	96.48	96.20	96.32	96.29
Allowed (%)	3.88	3.52	3.80	3.68	3.71
Disallowed (%)	0.00	0.00	0.00	0.00	0.00

	S-GSAS/6P: H18:R1-32 3:3:3 one RBD rotated (EMD-60109, PDB 8ZHN)	S1:H18:R1-32 1:1:1 (EMD-60110, PDB 8ZHO)	S1:H18:R1-32 2:2:2 (EMD-60111, PDB 8ZHP)
**Data collection and processing**			
Magnification	130,000	130,000	130,000
Voltage (kV)	300	300	300
Electron exposure ($e^{-}/Å^2$)	50	50	50
Defocus range (μm)	0.6-2.0	0.6-2.0	0.6-2.0
Pixel size (Å)	0.93	0.93	0.93
Movies (no.)	9,861	9,861	9,861
Initial particle images (no.)	3,335,676	3,335,676	3,335,676
Symmetry imposed	*C1*	*C1*	*C2*
Final particle images (no.)	13,185	178,003	144,598
Map resolution (Å)	4.13	3.57	3.68
FSC threshold	0.143	0.143	0.143
Map resolution range (Å)	3.67-21.44	3.44-10.00	3.53-16.12
**Refinement**			
Initial model used	PDB 7YEG	PDB 8HC5	PDB 8HC5
Model resolution (Å)	4.4	3.9	4.0
FSC threshold	0.5	0.5	0.5
Map sharpening *B* factor ($Å^2$)		-74	-75.5
Model composition			
Non-hydrogen atoms	45,309	11,429	22,856
Protein residues	5,829	1,498	2,998
Ligands	57	1	2
B factors ($Å^2$)			
Protein	162.70	195.18	182.59
Ligand	134.02	74.48	37.02
R.m.s. deviations			
Bond lengths (Å)	0.003	0.002	0.002
Bond angles (°)	0.628	0.518	0.525
**Validation**			
MolProbity score	1.63	1.42	1.55
Clashscore	6.37	4.00	5.80
Poor rotamers (%)	0.46	0.31	0.04
Ramachandran plot			
Favored (%)	95.95	96.49	96.49
Allowed (%)	4.05	3.51	3.51
Disallowed (%)	0.00	0.00	0.00

Supplementary Table 3 | Kinetic parameters of the recombinant mAbs binding to RBDs of SARS-CoV-2, SARS-CoV-1, Pangolin CoV GD1 and Bat CoV RaTG13 in Figs. 5a,b and S11

	SARS-CoV-2			SARS-CoV-1			Pangolin CoV GD1			Bat CoV RaTG13		
	k_{on} (M^{-1} s^{-1})	k_{off} (s^{-1})	K_D (nM)	k_{on} (M^{-1} s^{-1})	k_{off} (s^{-1})	K_D (nM)	k_{on} (M^{-1} s^{-1})	k_{off} (s^{-1})	K_D (nM)	k_{on} (M^{-1} s^{-1})	k_{off} (s^{-1})	K_D (nM)
**H4**	3.51´ 10^{5}	1.05´ 10^{-2}	29.95	-	-	no binding	2.88´ 10^{4}	1.02´ 10^{-2}	355	2.67´ 10^{4}	1.34´ 10^{-2}	503
**H5**	1.29´ 10^{5}	1.95´ 10^{-1}	1504	-	-	no binding	-	-	no binding	-	-	no binding
**H14**	5.36´ 10^{5}	1.82´ 10^{-2}	34.05	-	-	no binding	4.55´ 10^{4}	2.05´ 10^{-2}	451	4.99´ 10^{4}	1.51´ 10^{-2}	302
**H16**	5.09´ 10^{5}	4.98´ 10^{-2}	97.9	-	-	no binding	3.44´ 10^{4}	2.54´ 10^{-2}	737	-	-	no binding
**H18**	3.16´ 10^{5}	9.31´ 10^{-3}	29.49	1.23´ 10^{4}	2.83´ 10^{-3}	231	4.65´ 10^{4}	6.24´ 10^{-3}	134	1.84´ 10^{4}	6.12´ 10^{-3}	332

Supplementary Table 4 | Kinetic parameters of R1-26, H18 and S2A4 binding to the generated RBD mutants in Fig. 6c

	R1-26			H18			S2A4		
	k_{on} (M^{-1} s^{-1})	k_{off} (s^{-1})	K_D (nM)	k_{on} (M^{-1} s^{-1})	k_{off} (s^{-1})	K_D (nM)	k_{on} (M^{-1} s^{-1})	k_{off} (s^{-1})	K_D (nM)
**Wuhan-Hu-1**	5.07´ 10^{5}	2.91´ 10^{-3}	5.75	5.60´ 10^{5}	1.62´ 10^{-2}	28.99	3.80´ 10^{5}	9.60´ 10^{-3}	25.27
**Omicron**	-	-	no binding	-	-	no binding	-	-	no binding
**Omicron-L371S**	-	-	no binding	-	-	no binding	-	-	no binding
**Omicron-P373S**	-	-	weak binding	-	-	no binding	-	-	weak binding
**Omicron-F375S**	-	-	no binding	-	-	no binding	-	-	no binding
**Omicron-(L371S+P373S)**	1.36´ 10^{5}	2.31´ 10^{-3}	16.94	1.71´ 10^{5}	2.84´ 10^{-2}	166	1.07´ 10^{5}	9.58´ 10^{-3}	89.39
**Omicron-(P373S+F375S)**	1.59´ 10^{6}	7.85´ 10^{-2}	49.34	1.31´ 10^{6}	7.78´ 10^{-2}	59.56	7.07´ 10^{5}	2.73´ 10^{-2}	38.62
**Omicron-(L371S+F375S)**	4.81´ 10^{5}	9.49´ 10^{-2}	197	3.82´ 10^{5}	1.53´ 10^{-1}	400	6.31´ 10^{5}	4.75´ 10^{-2}	75.24
**Omicron-(L371S+P373S+F375S)**	7.27´ 10^{5}	1.64´ 10^{-3}	2.25	8.39´ 10^{5}	1.63´ 10^{-2}	19.39	7.32´ 10^{5}	7.51´ 10^{-3}	10.26

Supplementary References

1 Wrobel, A. G. *et al.* SARS-CoV-2 and bat RaTG13 spike glycoprotein structures inform on virus evolution and furin-cleavage effects. *Nat Struct Mol Biol* **27**, 763-767, doi:10.1038/s41594-020-0468-7 (2020).

2 Yuan, M. *et al.* A highly conserved cryptic epitope in the receptor binding domains of SARS-CoV-2 and SARS-CoV. *Science* **368**, 630-633, doi:10.1126/science.abb7269 (2020).

3 Xiong, X. *et al.* A thermostable, closed SARS-CoV-2 spike protein trimer. *Nature Structural & Molecular Biology* **27**, 934-941, doi:10.1038/s41594-020-0478-5 (2020).

4 Lan, J. *et al.* Structure of the SARS-CoV-2 spike receptor-binding domain bound to the ACE2 receptor. *Nature* **581**, 215-220, doi:10.1038/s41586-020-2180-5 (2020).
