# Supplementary material for: Antibodies utilizing VL6-57 light chains target a convergent cryptic epitope on SARS-CoV-2 spike protein and potentially drive the genesis of Omicron variants
Source: Nat Commun. 2024 Aug 31;15:7585. doi: 10.1038/s41467-024-51770-3 (PMC11366018; doi:10.1038/s41467-024-51770-3)
Supplement: Supplementary file 3 — Reporting Summary [file 41467_2024_51770_MOESM3_ESM.pdf]

nature portfolio

Corresponding author(s): Xiaoli Xiong

Last updated by author(s): Jul 10, 2024

Reporting Summary

Nature Portfolio wishes to improve the reproducibility of the work that we publish. This form provides structure for consistency and transparency in reporting. For further information on Nature Portfolio policies, see our Editorial Policies and the Editorial Policy Checklist.

Statistics

For all statistical analyses, confirm that the following items are present in the figure legend, table legend, main text, or Methods section.

n/a	Confirmed	
☐	☒	The exact sample size (n) for each experimental group/condition, given as a discrete number and unit of measurement
☐	☒	A statement on whether measurements were taken from distinct samples or whether the same sample was measured repeatedly
☐	☒	The statistical test(s) used AND whether they are one- or two-sided<br>*Only common tests should be described solely by name; describe more complex techniques in the Methods section.*
☒	☐	A description of all covariates tested
☒	☐	A description of any assumptions or corrections, such as tests of normality and adjustment for multiple comparisons
☐	☒	A full description of the statistical parameters including central tendency (e.g. means) or other basic estimates (e.g. regression coefficient) AND variation (e.g. standard deviation) or associated estimates of uncertainty (e.g. confidence intervals)
☐	☒	For null hypothesis testing, the test statistic (e.g. F, t, r) with confidence intervals, effect sizes, degrees of freedom and P value noted<br>*Give P values as exact values whenever suitable.*
☒	☐	For Bayesian analysis, information on the choice of priors and Markov chain Monte Carlo settings
☒	☐	For hierarchical and complex designs, identification of the appropriate level for tests and full reporting of outcomes
☒	☐	Estimates of effect sizes (e.g. Cohen's d, Pearson's r), indicating how they were calculated

Our web collection on statistics for biologists contains articles on many of the points above.

Software and code

Policy information about availability of computer code

Data collection	SerialEM version 3.8.7 and EPU automated image acquisition software;<br>Fortebio Octet RED96 instrument software (available and referenced in the Methods section)
Data analysis	Binding and neutralization data were analyzed using Graphpad Prism v8.0 as described in Methods.<br>CryoEM data processing (all available and referenced in methods): RELION v4.0; cryoSPARC v3.3.2/v4.2.0.<br>Modelling/structure refinement/visualization (all available and referenced in methods): Coot v0.9.6; PHENIX v1.20.1; UCSF Chimera v1.14; QtPISA v2.1.0.<br>Kinetic constants calculating: Fortebio Octet Data Analysis Software HT v12.0.2.59.<br>Polyreactivity data analysis: Graphpad Prism v8.0.<br>Sequence alignment: IMGT/V-QUEST (http://imgt.org).<br>Analysis of germline gene usage and CDR3 length were perform using the built-in function in R platform v4.2.<br>CDR3 amino acid composition analysis was performed using the R package ggseqlogo v0.1<br>The divergence from germline genes of all IgH or IgL sequences was equal to somatic hypermutations, and sequence identity to queried sequence was calculated using the R package Biostrings v2.60.2

For manuscripts utilizing custom algorithms or software that are central to the research but not yet described in published literature, software must be made available to editors and reviewers. We strongly encourage code deposition in a community repository (e.g. GitHub). See the Nature Portfolio guidelines for submitting code & software for further information.

Data

Policy information about availability of data

All manuscripts must include a data availability statement. This statement should provide the following information, where applicable:

- Accession codes, unique identifiers, or web links for publicly available datasets
- A description of any restrictions on data availability
- For clinical datasets or third party data, please ensure that the statement adheres to our policy

Databases used in this study include antibody repertoire databases from 3 previously datasets under accession numbers PRJCA003775, PRJCA007067 and PRJCA017560 (https://bigd.big.ac.cn/) (Niu et al., 2020; Yan et al., 2021; Zhang et al., 2022) and single-B V(D)J sequences from 5 previously datasets under accession numbers GSE230227, GSE171703, GSE158038, GSE158055; http://www.microbiome-bigdata.com/project/SARS-CoV-2/; and PRJCA012020 (https://bigd.big.ac.cn/) (Barmada et al., 2023; Dugan et al., 2021; Ferreira-Gomes et al., 2021; Ren et al., 2021; Zhang et al., 2020).
COV-AbDab database: http://opig.stats.ox.ac.uk/webapps/covabdab/.
Cryo-EM density maps for the structures of R1-26 or H18 Fab in complex with S-trimer or S1 fragment have been deposited in the Electron Microscopy Data Bank (EMDB) under accession codes EMD-60099, EMD-60100, EMD-60101, EMD-60102, EMD-60103, EMD-60104, EMD-60105, EMD-601066, EMD-60107, EMD-60108, EMD-60109, EMD-60110, EMD-60111. Related atomic models have been deposited in the Protein Data Bank (PDB) under accession codes 8ZHD, 8ZHE, 8ZHF, 8ZHG, 8ZHH, 8ZHI, 8ZHJ, 8ZHK, 8ZHL, 8ZHM, 8ZHN, 8ZHO, 8ZHP, respectively.

Research involving human participants, their data, or biological material

Policy information about studies with human participants or human data. See also policy information about sex, gender (identity/presentation), and sexual orientation and race, ethnicity and racism.

Reporting on sex and gender	Not applicable
Reporting on race, ethnicity, or other socially relevant groupings	Not applicable
Population characteristics	Not applicable
Recruitment	Not applicable
Ethics oversight	Not applicable

Note that full information on the approval of the study protocol must also be provided in the manuscript.

Field-specific reporting

Please select the one below that is the best fit for your research. If you are not sure, read the appropriate sections before making your selection.

☒ Life sciences ☐ Behavioural & social sciences ☐ Ecological, evolutionary & environmental sciences

For a reference copy of the document with all sections, see nature.com/documents/nr-reporting-summary-flat.pdf

Life sciences study design

All studies must disclose on these points even when the disclosure is negative.

Sample size	No sample size calculation was performed as this study does not involve human or animal subjects.
Data exclusions	No data were excluded.
Replication	All experiments were performed with appropriate replicates, as described in the figure legends and Methods section.
Randomization	Not applicable or relevant, as no human or animal subjects were used in this study.
Blinding	Blinding was not necessary in this study since no subjective allocation was involved.

Reporting for specific materials, systems and methods

We require information from authors about some types of materials, experimental systems and methods used in many studies. Here, indicate whether each material, system or method listed is relevant to your study. If you are not sure if a list item applies to your research, read the appropriate section before selecting a response.

Materials & experimental systems

n/a	Involved in the study	
☐	☒	Antibodies
☐	☒	Eukaryotic cell lines
☒	☐	Palaeontology and archaeology
☒	☐	Animals and other organisms
☒	☐	Clinical data
☒	☐	Dual use research of concern
☒	☐	Plants

Methods

n/a	Involved in the study	
☒	☐	ChIP-seq
☒	☐	Flow cytometry
☒	☐	MRI-based neuroimaging

Antibodies

Antibodies used	SARS-CoV-2 S2 polyclonal antibody (Sino Biological, cat. 40590-T62, dilution 1:2500);<br>HRP-labeled Goat Anti-Rabbit IgG(H+L) (Beyotime, cat. A0208, dilution 1:2500).<br>HRP-labeled goat anti-human IgG(H+L) (Jackson ImmunoResearch Laboratories, cat. 609-035-213, dilution 1:5000)
Validation	All antibodies are commercially available and validated by the manufacturers or by refereed articles cited on each supplier's website. The information are included in the Methods section.

Eukaryotic cell lines

Policy information about cell lines and Sex and Gender in Research

Cell line source(s)	Expi293F: Thermo Fisher Scientific, A14527;<br>HEK 293T: ATCC, CRL-3216;<br>Vero E6: ATCC, CRL-1586.
Authentication	All cell lines were frequently checked for growth rates, cellular morphologies and functions, but none of cell lines were authenticated.
Mycoplasma contamination	The cell lines were not contaminated by mycoplasma as determined by using the Lonza Mycoplasma Detection Kit.
Commonly misidentified lines (See ICLAC register)	No commonly misidentified cell lines were used.
